# Triple-negative breast cancer

**DOI:** 10.1186/bcr2574

**Published:** 2010-10-22

**Authors:** Reinaldo D Chacón, María V Costanzo

**Affiliations:** 1 Oncology Department, Instituto Alexander Fleming, Cramer 1180, zip code 1426 ANZ, Ciudad Autonoma de Buenos Aires, Argentina

## Abstract

Perou's molecular classification defines tumors that neither express hormone receptors nor overexpress HER2 as triple-negative (TN) tumors. These tumors account for approximately 15% of breast cancers. The so-called basaloid tumors are not always synonymous with TN tumors; they differ in the fact that they express different molecular markers, have a higher histologic grade, and have a worse prognosis. Clinically they occur in younger women as interval cancer, and the risk of recurrence is higher within the first 3 years. Distant recurrences in the brain and visceral metastases are more common than in hormone receptor-positive tumors. Therapeutically, despite being highly chemosensitive, their progression-free time is generally short. In terms of chemotherapeutic treatment, anthracyclines and taxanes are useful drugs, and high response rates have been described for the combination of ixabepilone-capecitabine and platinums. The combination with antiangiogenic drugs has also proven useful. A group of new drugs, poly-(ADP-ribose)-polymerase inhibitors, showed favorable results in TN tumors with BRCA mutation. There are currently several ongoing studies with new drugs including epidermal growth factor receptor inhibitors, c-kit inhibitors, Raf/Mek/Map kinase inhibitors and mTOR inhibitors.

## Introduction

Breast cancer undoubtedly constitutes what is expected from a large proportion of the other neoplasms: a group of diseases characterized by different morphologies, biological behaviors, forms of presentation and clinical evolution. This suspicion, based on different responses to the same treatment, would gradually become clearer through findings such as hormone receptors (HRs) and, most recently, the HER family, along with the description of metabolic chains and genetic variations (mutation, deletion or overexpression), all of which gave rise to specific targets whose optimal use is continually under study.

The introduction of HRs in clinical routine use not only showed the usefulness of endocrine therapy in HR-positive cases (60 to 80%) but also the special aggressiveness of HR-negative cases. Even today, estrogen receptors (ERs) are likely to be one of the most important prognostic and, naturally, predictive factors (their negativity calls for the use of chemotherapeutic agents, in contrast to hormone therapy use when they are positive). From a practical standpoint, the concept of negativity has been generalized as lack of expression of both ER and progesterone receptor. HR-negative tumors are accompanied by a high histologic grade. p53 is mutated in up to 82% of basal-like breast carcinomas [1] by gene expression analysis as well as protein expression analysis. This phenotype is also particularly associated with BRCA1 mutations [2].

The significance of HER2 amplification or overexpression was recognized in 1987 [3]; it characterizes about 20% of breast tumors and is usually seen in HR-negative tumors, with a higher percentage of recurrences and mortality rates [4]. The standard use of HER2 assessment (around 1999) led to the recognition of a subgroup with worse prognosis and, at the same time, to the development of specific molecules, of which trastuzumab was the first [5]. HER2 overexpression also identified tumors with estrogen-negative, progesterone-negative receptors and HER2-negative receptors. The tumors with estrogen-negative, progesterone negative and HER2-negative are known as triple-negative (TN) tumors and account for about 15% of breast tumors [6,7].

The molecular classification described by Perou and colleagues showed, through the gene expression profile, remarkable differences between HR-positive tumors and HR-negative tumors [8]. The former were classified as luminal tumors (luminal A or luminal B based on their higher or lower receptor expression), and the latter were divided into three subgroups: tumors with HER2 amplification; basaloid tumors, resembling normal basal or myoepithelial cells; and tumors with loss of HR, of HER2 amplification and of basaloid characteristics (which show molecular similarity with normal mammary stromal cells).

## Basaloid and triple-negative tumors

Table 1 presents basaloid and TN tumor incidence rates taking into account HR and HER2 phenotypic expression and the basaloid variant from the molecular classification. A common assumption is that basaloid tumors and TN tumors are the same entity – based on the fact that the former are usually TN tumors, thus assuming that the TN phenotype includes basaloid tumors. Table 2 presents general characteristics of basaloid tumors [9,10].

**Table 1:** Breast cancer: basaloid and triple-negative tumor incidence rates

Tumor type	Incidence rate
Positive hormone receptors	50 to 80%
HER2-positive	20 to 25%
Triple negative	12 to 20%
Basaloid^a^	15%

Rates taking into account hormone receptor and HER2 phenotypic expression and the basaloid variant from the molecular classification. ^a^39% occur in premenopausal African-American versus Caucasian women of any age.

**Table 2:** Breast cancer: general characteristics of basaloid tumors

Strong cytokeratin 5/6, cytokeratin 14, and cytokeratin 17 expression
Negative hormone receptor tumors, with low expression of HER2
May express epidermal growth factor receptors and c-kit receptors
High histologic grade and worse prognosis than nonbasaloid triple-negative tumors
BRCA1 mutation

In a recently published series, 10% of basaloid tumors were HER2-positive, 12% were ER-positive, 84% were histologic grade III, most tumors were >2 cm and 40% had positive axillary nodes [11]. On the other hand, there are many publications that show differences in the molecular profile of basaloid tumors and TN tumors [12-14]. Correct identification of each subgroup would explain the mixed treatment outcomes and will aid the search for specific targets. Finally, it is worth noting that TN tumors include different histological variants (for example, infiltrating ductal, medullary, squamous, apocrine). The association between TN tumors and BRCA1 [15,16] is presented in Table 3.

**Table 3:** Breast cancer: association between triple-negative tumors and BRCA1

Association	Incidence
Tumors with BRCA1 mutation are triple-negative tumors	90%
BRCA1 tumors are basaloid tumors	80 to 90%
Triple-negative tumors are tumors with BRCA1 mutations	10%

## Triple-negative tumors, clinical expression and recurrence patterns

The general characteristics of TN tumors are presented in Table 4, some of which are unique clinical features. TN tumors often present as interval cancer [17] and, in turn, are detected more frequently through clinical examination than with a mammogram or an ultrasound [18], which is suggestive of rapid growth and tissue density similar to normal tissue. Even small-size tumors present a high incidence of lymph node involvement [11].

**Table 4:** General characteristic of triple-negative breast cancers

Often present as interval cancer
Weak association between tumor size and lymph node involvement
High risk of early recurrence
Peak recurrence rate is seen between the first and third years after diagnosis
Metastases are rarely preceded by local recurrence
Local recurrence is not predictive of metastatic disease
More prevalent in young women
Stronger association with obesity
Higher prevalence of brain metastases
Most deaths occur in the first 5 years
Rapid progression from the onset of metastasis to death
Highly chemosensitive
Risk factor in tumors with negative axillary nodes
Specific target molecules have only been determined recently

Follow-up of about 200 patients diagnosed with TN in Toronto between 1987 and 1997 showed a peak of recurrence rate much greater than that of nontriple-negative (nTN) tumors during the first and third years, as well as a higher 5-year mortality rate [18]. This was subsequently confirmed in patients treated with neoadjuvant therapy at M.D. Anderson [19], who showed a higher 3-year relapse and mortality rates. Dent and colleagues found few cases in which local recurrence preceded distant metastases [18]; these, in turn, are more common in the viscera and soft tissues than in bone, while bone metastases are a common pattern in luminal tumors [20,21].

Basaloid tumors are characterized by lung and brain relapse, with the addition of the liver for TNs in general. Brain involvement is also more common in HER2-positive tumors, but in these cases – unlike TN tumors – the specific (anti-HER2) therapies available to control the other metastatic sites allow for longer survival [22]. The higher prevalence in young women [23] may be partially related to BRCA1-mutated basaloid tumors and, apparently, to parity and age at first full-term pregnancy, as well as to breastfeeding time. All of the above has been more commonly observed in young African American women [24]. Obesity as an independent variable in TN tumors seems to be associated with worse prognosis [25].

About the outcomes seen with conservative surgery in TN tumors, observations from retrospective studies show small differences with nTN tumors [26]. The high chemosensitivity of these tumors as well as their poor prognosis, which will be described later, are striking. The 2010 National Clinical Cancer Network Guidelines [27] do not recommend adjuvant chemotherapy in TN tumors for T1aN0 tumors; adjuvant chemotherapy is considered for T1bN0 tumors and is suggested for T1cN0 tumors.

## Current therapeutic options

### Available therapies

#### Chemotherapy

**Figure 1:**
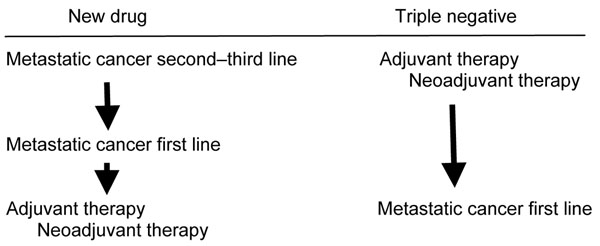
**Triple-negative tumors.** Reverse burden of proof.

One of the characteristics of TN tumors is their high chemosensitivity, but with a short time to progression and survival. The use of certain drugs in the metastatic setting led to the retrospective outcome analysis in the adjuvant and neoadjuvant settings, which was subsequently applied to metastatic disease (reverse burden of proof; Figure 1). The recent appearance of poly-(ADP-ribose)-polymerase (PARP) 1 leads back to the original model, but as first-line therapy since there is no standard chemotherapeutic treatment.

Review of TN tumor subgroups in adjuvant therapy studies, in the case of CALGB 9344 (patients with positive axillary nodes to compare the addition of pacli-taxel to different anthracycline doses), shows significant benefits (*P* = 0.002) for this combination, although the benefits were independent of HER2 status [28]. For the same kind of combination – but instead comparing paclitaxel every 21 days versus paclitaxel once a week after four courses of adriamycin-paclitaxel every 3 weeks – Loesch and colleagues showed statistically significant results (*P* = 0.037) in 378 TN patients treated with paclitaxel once a week [29]. A previous Intergroup study (C9741) had found differences in favor of dose density with adriamycin and paclitaxel in patients with negative ERs, but not in ER-positive patients [30]; this highlights the importance of chemotherapy in hormone-independent tumors.

Several studies on neoadjuvant therapy show the importance of chemotherapy in TN tumors. Rouzier and colleagues assessed chemosensitivity in 82 patients based on the molecular classification using the anthracycline and taxane combination, and found a 45% rate of complete pathological remission (cPR) for HER2-positive and basaloid tumors, versus 6% for luminal tumors [31]. Similarly, but using the anthracycline and cyclophosphamide combination in 107 immunohistochemically defined patients, Carey and colleagues observed overall response rates of 70% (HER2^+^), 85% (basaloid), and 47% (luminal) [32]. The difference was much greater when cPR was considered: 36%, 27%, and 7%, respectively.

Liedtke and colleagues considered 1,118 patients who received neoadjuvant therapy at M.D. Anderson between 1985 and 2004, among which there were 255 TN tumors and 868 nTN tumors [19]. It should be noted that trastuzumab was not used and that a 10% cut-off point was used to define negative ERs. The cPR percentages (about 22%) favored TN tumors significantly both for anthracyclines combined with taxanes or not, but the most suggestive detail was the similar disease-free time for patients with cPR, either with TN tumors or nTN tumors. On the other hand, patients with TN tumors who did not achieve cPR had a poor outcome compared with women with nTN tumors (3-year disease-free rate, 68% vs. 88%; *P* = 0.0001). Recently, in a phase II study using ixabepilone monotherapy, a 26% cPR rate was found for breast tumor, and a 19% cPR rate when axillary involvement was included [33].

The use of anthracyclines and taxanes in breast cancer metastatic disease has shown greater efficacy in patients with negative ERs; based on these results, both drug families are indicated as first-line treatment for TN tumors. Certain difficulties should be considered, however: these drugs are commonly used for adjuvant therapy, maximum anthracycline doses are cardiotoxic, and the disease-free time is short – all of which cast doubts about chemosensitivity to these drugs. The mean duration of chemotherapy response was approximately 12 weeks for the first-line treatment, 9 weeks for the second-line treatment and only 4 weeks for the third-line treatment in an analysis of 111 patients with TN tumors [34]. Likewise, in terms of survival-related variables in addition to those already known for nTN tumors (previous adjuvant therapy, metastasis type, and so forth), age >50 years implied a better survival, as opposed to what is observed in nTN patients.

An early study on the use of ixabepilone plus capeci-tabine versus capecitabine monotherapy [35] in patients who failed to anthracyclines plus taxanes showed a higher response rate (27% vs. 9%) and a longer time to progression (4.1 months vs. 2.1 months) for the combination in the TN subgroup. Subsequently, and using the same comparison, the pooled results of the 046 study (taxane resistant) and the 048 study (population pretreated with anthracyclines and taxanes) were presented at the 2008 San Antonio Breast Cancer Symposium [36]. Benefits were found for the ixabepilone-capecitabine combination in terms of objective responses (31% vs. 15%) and time to progression (4.2 months vs. 1.7 months), but not for overall survival (10.3 months vs. 9.0 months). The ongoing adjuvant study PACS-08, which stratifies TN tumors, includes the use of ixabepilone in one of its arms.

The role of platinums was re-considered in TN tumors taking into account their mechanism of action and the potential DNA changes in these tumors, which are phenotypically and molecularly similar to BRCA1 tumors. DNA repair defects may be adequate targets for alkylating agents [37-39]. In a phase II study, Garber and colleagues showed a 21% cPR with a neoadjuvant platinum-based regimen [40]; and Sirohi and colleagues, using different platinum-based regimens, showed higher chemosensitivity in TNs compared with nTNs, both in the neoadjuvant and metastatic settings, but curiously enough also showed a survival advantage in the metastatic setting [41]. Other studies did not show such difference, but rather the opposite [42,43]. There are many ongoing trials in the adjuvant, neoadjuvant and metastatic settings: carboplatin versus docetaxel (NCT00532727), four cycles of epirubicin and cyclophosphamide followed by four cycles of docetaxel alone or combined with carboplatin (NCT00432173), gemcitabine + cisplatin (NCT00601159), and gemcitabine + oxaliplatin (NCT00674206) are some examples [44]. The results obtained with PARP1 inhibitors will probably modify some of the combinations, but platinums will most likely remain useful.

#### Bevacizumab

Angiogenesis is one of the mechanisms of breast cancer progression, and even though vascular endothelial growth factor overexpression has not been found, basaloid tumors show glomeruloid microvascular proliferation [45].

The monoclonal antibody bevacizumab was approved by the US Food and Drug Administration as first-line treatment in metastatic breast cancer in combination with paclitaxel, as it showed benefits compared with paclitaxel monotherapy in terms of response rates (36.9% vs. 21.2%) and time to progression (8.8 months vs. 4.6 months). This phase III study (E2100) included a vast majority of HER2-negative patients (91%) and the TN subgroup also showed clear advantages with the addition of bevacizumab [46]. Two additional studies demonstrated increased objective response rates with the addition of bevacizumab in metastatic cancer: the phase III study AVADO combined bevacizumab with docetaxel [47], and the RIBBON-1 study used bevacizumab in combination with different drugs (capecitabine, nab-paclitaxel, docetaxel or anthracyclines) [48].

Based on these results, there are now ongoing protocols that have included this monoclonal antibody in different adjuvant chemotherapy regimens in only TN tumors (NCT00528567 BEATRICE) or only HER2-negative tumors (CALGB 40603), as well as phase II trials in TN patients in the neoadjuvant and metastatic settings [44].

### Therapies under study

#### Antiangiogenic therapy

Sunitinib – a tyrosine-kinase inhibitor whose targets include vascular endothelial growth factors 1, 2 and 3, platelet-derived growth factors alpha and beta, c-KIT and colony-stimulating factor 1 [49-51] – showed anti-tumor activity in several preclinical studies with breast cancer models, both alone or in combination with chemotherapeutic agents. In 64 pretreated patients (20 with TN tumors), 61 of whom were treated with anthracyclines and taxanes, Burstein and colleagues reported seven partial responses, of which three were in TN tumors [52]. A phase III randomized study evaluated sunitinib versus capecitabine in patients with previously treated HER2-negative advanced breast cancer [53]. More than 30% of the patients had TN disease and less than two prior regimens for metastatic disease. The primary end point, disease-free survival, was not met; indeed, the median disease-free survival was better with capecitabine therapy (4.2 months vs. 2.8 months). No statistically significant difference in overall survival was noted. After these results, the Independent Data Monitoring Committee recommended stopping trial enrollment for futility. Sunitinib cannot be recommended as monotherapy on this dosing schedule for treatment of advanced metastatic breast cancer.

Sorafenib is a potent multikinase inhibitor with antiangiogenic and antiproliferation activity. This inhibitor is indicated for the treatment of advanced renal cell carcinoma and unresectable hepatocellular carcinoma. As a single agent, sorafenib has shown modest activity in patients with advanced breast cancer. Two phase IIb trials evaluating efficacy and safety of sorafenib with chemotherapy or placebo were presented at the San Antonio Breast Cancer Symposium 2009 [54,55].

The SOLTI-0701 trial evaluated the combination of sorafenib (400 mg twice daily) with capecitabine or placebo in patients with metastatic breast carcinoma (first or second line). Thirty percent of patients had TN disease. Median progression-free survival was extended in patients treated with the combination of sorafenib-capecitabine in comparison with the combination sorafenib-placebo. These results were statistically significant (hazard ratio, 0.57; *P* = 0.0006). The incidence of grade III hand-foot was 45% versus 13% in the placebo group [54].

The second trial evaluated sorafenib in combination with paclitaxel or placebo, as first-line therapy in patients with locally recurrent or metastatic breast cancer. Forty percent of patients had TN disease. The hazard ratio for progression-free survival was 0.78 (*P* = 0.08). The incidence of grade III hand-foot syndrome was 30% versus 3% in the placebo group, a trend favoring the sorafenib-paclitaxel group. The concerning toxicity was the grade III hand-foot syndrome. The study presenters called these rates unacceptable, and recommend carefully monitoring patients for the occurrence of the early stages of hand-foot toxicity and dose-reducing more aggressively to reduce these events rates [55]. A somewhat lower dose of sorafenib may be utilized as a means of reducing the hand-foot toxicity in phase III trials.

#### Poly-(ADP-ribose)-polymerase inhibitors

Frequently, in different situations, cell DNA can be damaged. This is the reason why repair mechanisms come into play, of which PARP – particularly PARP1 – plays a vital role together with other mechanisms that involve BRCA1 and BRCA2. Mutations in any of the BRCA alleles are associated with a higher cancer risk, including breast cancer, ovarian cancer and prostate cancer. In the case of PARP1 inhibition and the resulting damage to one of the DNA arms, and in the absence of homologous recombination due to abnormal BRCA, so-called synthetic lethality occurs [56]. *In vitro* BRCA1-deficient or BRCA2-deficient cells were shown to be 1,000 times more sensitive to PARP inhibition than normal cells [37,57,58].

Fong and colleagues recently published their results using olaparib (AZD2281), an oral PARP inhibitor [56]. The study enrolled 60 patients, of which 22 were BRCA1 or BRCA2 mutation carriers, and one patient had family history of tumors related to these mutations. Except for two of these patients with an atypical location (small-cell lung cancer and vaginal adenocarcinoma) who progressed quickly, 12 of the 19 remaining patients (63%) experienced clinical benefit. None of the patients without the mutation showed response. Of the nine breast cancer patients, two BRCA2 mutation carriers achieved clinical response (one with complete remission and the other with stable disease for 7 months). Eight out of 21 patients with ovarian cancer responded to olaparib therapy.

Prior to the previous publication, two presentations at the American Society of Clinical Oncology 2009 showed the results achieved with PARP1 inhibitors. In a phase II study comparing two doses of olaparib (100 mg vs. 400 mg) in 54 breast cancer patients with BRCA mutation and most of them resistant to taxanes and anthracyclines, divided into two groups, Tutt and colleagues observed 41%, 4% and 5.7 months for objective remission, complete remission and time to progression, respectively, with the 400 mg dose, and 22%, 0% and 3.8 months, respectively, with the 100 mg dose [59]. It is worth noting that 2/3 of patients treated with the 400 mg dose had a BRCA1 mutation.

The other presentation addressed the concept of DNA molecule damage caused by chemotherapeutic agents associated with a PARP1 inhibitor; in this case, intravenous BSI-201 [60]. Population characteristics included TN breast cancer with two or fewer previous treatment regimens, of which 59 patients received a carboplatin-gemcitabine regimen and 57 patients the same chemotherapy regimen plus BSI-201. The combination showed greater clinical benefit (52% vs. 12%), progression-free time (6.9 months vs. 3.3 months, *P* = 0.0001) and overall survival (9.2 months vs. 5.7 months, *P* = 0.0005).

Other PARP inhibitors are being studied; for example, AGO 14699 in locally advanced or metastatic breast cancer and BRCA1/2-mutated ovarian cancer, and AZD2881 in BRCA1/2-mutated ovarian cancer and metastatic TN or BRCA-mutated breast cancer. In a phase I study, AZD2881 was combined with carboplatin to treat metastatic breast cancer or BRCA-mutated ovarian cancer. The impressive phase II results with the PARP inhibitors have led to a definitive phase III study involving more than 420 patients that will be finished in 2010.

## Other targeted therapies

### Epidermal growth factor receptor inhibition

Basal-like TN breast cancers express basal markers such as cytokeratin 5/6 and epidermal growth factor receptor.

Epidermal growth factor receptor mRNA is more commonly observed and is at higher levels in basaloid tumors (54%). This marker is a poor prognosis predictor regardless of axillary lymph node involvement and tumor size [61]. Given its diagnostic and prognostic role in basal-like TN breast cancer, epidermal growth factor receptor’s therapeutic role has been assessed with drugs that antagonize its action [62].

Cetuximab is a chimeric monoclonal antibody that inhibits the epidermal growth factor receptor. Some reports suggest cetuximab efficacy in TN breast cancer [63].

TBCRC 001 is a phase II study that randomized 102 patients with basaloid TN metastatic breast cancer to cetuximab alone, with carboplatin at progression (arm 1) or to initial cetuximab plus carboplatin (arm 2) [64]. The primary endpoint was the objective response. Fifty-four percent of patients had received prior chemotherapy for metastatic disease. While monotherapy was well tolerated, it showed poor activity: 6% with partial response, 4% achieved stable disease and 10% showed clinical benefit. On the contrary, the combined treatment showed higher rates of partial responses (18%) and clinical benefit (27% and 10% for stable disease). In line with the aggressive nature of these tumors, progression-free survival was 2 months.

Another phase II study randomized 165 patients with metastatic breast cancer to carboplatin and weekly irinotecan with/without cetuximab [65]. The subgroup of patients with TN tumors (72 patients) showed a higher response rate in the cetuximab arm (49% vs. 30%).

At present, several phase II studies are assessing different cetuximab combinations with chemotherapy in TN metastatic breast cancer: phase I–II with paclitaxel and phase II with cisplatin [66]. Other epidermal growth factor receptor inhibitors, such as gefitinib, did not show activity in this subgroup of patients [67]. Several clinical trials are currently assessing the efficacy of adding either a mAb, like cetuximab, or a tyrosine-kinase inhibitor, like erlotinib, in the treatment of TN breast cancer

### Src tyrosine kinase inhibitors

The Src tyrosine kinase (Rous sarcoma virus) is also over-expressed in breast cancer and is associated with metastatic disease progression [68,69]. Dasatinib is an oral, small-molecule tyrosine kinase inhibitor that acts on proteins src and abl. Preclinical studies show dasatinib’s activity to inhibit the growth of basal-like breast cancer cell lines [70,71], providing the rationale for clinical research in this specific subgroup. A phase II trial showed a clinical benefit rate of 9% in TN metastatic breast cancer, but discontinuation of therapy and dose reductions weakened the results [72]. There are currently several studies evaluating dasatinib as monotherapy or in combination regimens in this setting.

### mTOR inhibitors

mTOR (mammalian target of rapamycin) is a cell cycle regulator as well as an effector of the final common pathway of phosphatidylinositol 3-phosphate phosphatase and PTEN/AKT (tensin homolog deleted from chromosome 1). This metabolic pathway is damaged in breast cancer [70]. Loss of the PTEN tumor suppressor gene is common in TN breast cancer, which causes increased mTOR activation [73]. This would be the rationale for the use of mTOR inhibitors for this condition.

A phase II randomized study evaluates two everolimus (oral mTOR inhibitor) regimens for first-line or second-line treatment in 59 metastatic breast cancer patients, of which 20 patients are HER2 receptor-negative [74]. The regimens compared are 10 mg/day or 70 mg/week; a 12% response was observed in the daily regimen versus 0% in the weekly one; there was a higher incidence of pneumonitis in the daily regimen (16% vs. 6%) and no biological markers of effectiveness.

A phase II, nonrandomized study is evaluating temsirolimus (intravenous mTOR inhibitor) in TN metastatic breast cancer [66], and a phase III randomized study is evaluating everolimus in combination with anthracyclines and taxanes in the neoadjuvant setting.

### Heat shock protein 90 inhibitors

Heat shock protein 90 is a cellular chaperone protein that facilitates the post-translational maturation and stabilization of a number of conformationally labile client proteins, including steroid receptors, RAF-1, cyclin-dependent kinase 4, AKT and other proteins that play a role in transducing proliferative signals [75]. When heat shock protein 90 function is inhibited, their client protein is degraded by proteosomes.

Geldanamicyn and tanespimycin have demonstrated activity in HER2-positive metastatic breast cancer disease [76]. The inhibitor PU-H71 demonstrated impressive response in TN breast cancer disease in preclinical studies [77]*.*

## Future directions

TN breast cancer represents a unique subgroup, with a specific molecular profile, an aggressive behavior pattern, a relative lack of effective therapies and a poor prognosis.

A large number of therapies have been developed to date for specific molecular targets used as monotherapy or combined with traditional chemotherapy. Currently there are over 50 clinical trials assessing various therapeutic options. Improved knowledge of the role of BRCA1 and the discovery of metabolic pathways has led to the development of other therapeutic strategies. Finding new markers expressed in basaloid and TN tumors will allow for the use of other therapeutic targets, such as αβ-crystallin, Sox2, embryonic transcription factor, osteopontin, phosphorylated glycoprotein, nestin and type 4 intermediate filament protein. It is also necessary to develop research in the evaluation of predictive factors of treatment response. The assessment of caveolin 1 and caveolin 2 as a predictive marker of response to nab-paclitaxel, and of p63 and p73 as markers of platinum sensitivity is increasingly important.

Breast carcinomas have been reported to contain a subpopulation of CD44^+^/CD24^–^ tumor cells with stem-cell-like properties. The discovery of the CD44/CD24 phenotype and its relation with unfavorable prognosis in TN breast cancer disease also makes CD44 targeting an attractive therapeutic alternative [78]. This line of research will enable promotion of the use of specific targeted therapies and will allow progress in the development of an early treatment that may change the aggressive course of the disease.

## Abbreviations

cPR: complete pathological remission; ER: estrogen receptor; HR: hormone receptor; mAb: monoclonal antibody; nTn: nontriple negative; PARP: poly-(ADP-ribose)-polymerase; TN: triple negative.

## Competing interests

The authors declare that they have no competing interests.

Published: 22 October 2010

## References

[B1] Sørlie T Perou CM Tibshirani R Aas T Geisler S Johnsen H Hastie T Eisen MB van de Rijn M Jeffrey SS Thorsen T Quist H Matese JC Brown PO Botstein D Eystein Lønning P Børresen-Dale AL Gene expression patterns of breast carcinomas distinguish tumor subclasses with clinical implications Proc Natl Acad Sci USA 2001 98 10869 10874 11553815 10.1073/pnas.191367098 PMC58566

[B2] Lakhani S Van De Vijver M The patology of familial breast cancer: predictive value of immunohistochemical markers estrogen receptor, progesterone receptor, HER2, and p53 mutations in BRCA1 and BRCA2 J Clin Oncol 2002 20 2310 2318 10.1200/JCO.2002.09.023 11981002

[B3] Slamon DJ Clark GM Wong SG Levin WJ Ullrich A McGuire WL Human breast cancer: correlation of relapse and survival with amplification of the HER-2/neu oncogene Science 1987 235 177 182 10.1126/science.3798106 3798106

[B4] Wolff AC Hammond ME Schwartz JN Hagerty KL Allred DC Cote RJ Dowsett M Fitzgibbons PL Hanna WM Langer A McShane LM Paik S Pegram MD Perez EA Press MF Rhodes A Sturgeon C Taube SE Tubbs R Vance GH van de Vijver M Wheeler TM Hayes DF American Society of Clinical Oncology College of American Pathologists American Society of Clinical Oncology/College of American Pathologist guideline recommendation for human epidermal growth factor receptor 2 testing in breast cancer J Clin Oncol 2007 25 118 145 10.1200/JCO.2006.09.2775 17159189

[B5] Ross JS Fletcher JA Bloom KJ Linette GP Stec J Symmans WF Pusztai L Hortobagyi GN Targeted therapy in breast cancer: the HER-2/neu gen and protein Mol Cell Proteomics 2004 3 379 398 10.1074/mcp.R400001-MCP200 14762215

[B6] Sørlie T Perou CM Tibshirani R Aas T Geisler S Johnsen H Hastie T Eisen MB van de Rijn M Jeffrey SS Thorsen T Quist H Matese JC Brown PO Botstein D Eystein Lønning P Børresen-Dale AL Gene expression patterns of breast carcinomas distinguish tumor subclasses with clinical implications Proc Natl Acad Sci USA 2001 98 10869 10874 11553815 10.1073/pnas.191367098 PMC58566

[B7] Sorlie T Tibshirani R Parker J Hastie T Marron JS Nobel A Deng S Johnsen H Pesich R Geisler S Demeter J Perou CM Lønning PE Brwon PO Børresen-Dale AL Botstein D Repeated observation of breast tumor sub-types in independent gene expression data set Proc Natl Acad Sci USA 2003 100 8418 8423 12829800 10.1073/pnas.0932692100 PMC166244

[B8] Perou CM Sorlie T Eisen MB van de Rijn M Jeffrey SS Rees CA Pollack JR Ross DT Johnsen H Akslen LA Fluge O Pergamenchikov A Williams C Zhu SX Lønning PE Børreson-Dale AL Brown PO Botstein D Molecular portraits of human breast tumours Nature 2000 406 747 752 10.1038/35021093 10963602

[B9] Rakha EA El-Sayed ME Green AR Lee AH Robertson JF Ellis IO Prognostic markers in triple-negative breast cancer Cancer 2007 109 25 32 10.1002/cncr.22381 17146782

[B10] Bertucci F Finetti P Cervera N Esterni B Hermitte F Viens P Birnbaum D How basal are triple-negative breast cancer Int J Cancer 2008 123 236 240 10.1002/ijc.23518 18398844

[B11] Sotiriou C Phil D Pusztaj J Gene-expression signatures in breast cancer N Engl J Med 2009 360 790 800 10.1056/NEJMra0801289 19228622

[B12] Nielsen TO Hsu FD Jensen K Cheang M Karaca G Hu Z Hernandez-Boussard T Livasy C Cowan D Dressler L Akslen LA Ragaz J Gown AM Gilks CB van de Rijn M Perou CM Immunohistochemical and clinical characterization of the basal-like sub-type of invasive breast carcinoma Clin Cancer Res 2004 10 5367 5374 10.1158/1078-0432.CCR-04-0220 15328174

[B13] Livasy CA Karaca C Nanda R Tretiakova MS Olopade OI Moore DT Perou CM Phenotypic evaluation of the basal-like subtype of invasive breast carcinoma Mod Pathol 2006 19 264 271 10.1038/modpathol.3800528 16341146

[B14] Rakha EA Tan DS Foulkes WD Ellis IO Tutt A Nielsen TO Reis-Filho JS Are triple negative tumours and basal-like breast cancer synonymous? Breast Cancer Res 2007 9 404 18279542 10.1186/bcr1827 PMC2246182

[B15] Foulkes WD Stefansson IM Chappuis PO Bégin LR Goffin JR Wong N Trudel M Akslen LA Germline BRCA1 mutations and a basal epithelial phenotype in breast cancer J Natl Cancer Inst 2003 95 1482 1485 14519755 10.1093/jnci/djg050

[B16] Lakhani SR Reis-Filho JS Fulford L Penault-Llorca F van der Vijver M Parry S Bishop T Benitez J Rivas C Bignon YJ Chang-Cluade J Hamann U Cornelisse CJ Devilee P Beckmann MW Nestle-Krämling C Daly PA Haites N Varley J Lalloo F Evans G Maurgard C Meijers-Heijboer H Klijn JG Olah E Gusterson BA Pilotti S Radice P Schermeck S Sobol H Breast Cancer Linkage Consortium Prediction of BRCA1 status in patients with breast cancer using estrogen receptor and basal phenotype Clin Cancer Res 2005 11 5175 5180 10.1158/1078-0432.CCR-04-2424 16033833

[B17] Collett K Stefansonn IM Eide J Braaten A Wang H Eide GE Thoresen SØ Foulkes WD Akslen LA A basal epithelial phenotype is more frequent in interval breast cancer compared with screen detected tumors Cancer Epidemiol Biomarkers Prev 2005 14 1108 1112 10.1158/1055-9965.EPI-04-0394 15894660

[B18] Dent R Trudeau M Pritchard KL Hanna WM Kahn HK Sawka CA Lickley LA Rawlinson E Sun P Narod SA Triple-negative breast cancer: clinical features and patterns of recurrence Clin Cancer Res 2007 13 4429 4434 10.1158/1078-0432.CCR-06-3045 17671126

[B19] Liedtke C Mazouni C Hess KR André F Tordai A Mejia JA Symmans WF Gonzalez-Angulo AM Hennessy B Green M Cristofanilli M Hortobagyi GN Pusztai L Response to neoadyuvant therapy and long-term survival in patients with triple-negative breast cancer J Clin Oncol 2008 26 1275 1281 10.1200/JCO.2007.14.4147 18250347

[B20] Smid M Wang Y Zhang Y Sieuwerts AM Yu J Klijn JG Foekens JA Marten JW Subtypes of breast cancer show preferential site of relapse Cancer Res 2008 68 3108 3114 10.1158/0008-5472.CAN-07-5644 18451135

[B21] Lin NU Claus E Sohl J Razzak AR Arnaout A Winer EP Sites of distant recurrence and clinical outcomes in patients with metastatic triple-negative breast cancer: high incidence of central nervous system metastases Cancer 2008 113 2638 2645 18833576 10.1002/cncr.23930 PMC2835546

[B22] Bendell JC Domchek SM Burstein HJ Harris L Younger J Kuter I Bunnell C Rue M Gelman R Winer E Central nervous system metastases in women who receive trastuzumab-based therapy for metastasic breast carcinoma Cancer 2003 97 2972 2977 10.1002/cncr.11436 12784331

[B23] Bauer KR Brown M Cress RD Parise CA Caggiano V Descriptive analysis of estrogen receptor (ER)-negative, progesterone receptor (PR) negative, and HER-2 negative invasive breast cancer, the so-called triple-negative phenotype: a population-based study from the California Cancer Registry Cancer 2007 109 1721 1728 10.1002/cncr.22618 17387718

[B24] Morris GJ Naidu S Topham AK Guiles F Xu Y McCue P Schwartz GF Park PK Rosenberg AL Brill K Mitchell EP Differences in breast carcinoma characteristics in newly diagnosed African-American and Caucasian patients: a single-institution compilation compared with the National Cancer Institute’s Surveillance, Epidemiology, and End Results database Cancer 2007 110 876 884 10.1002/cncr.22836 17620276

[B25] Vona-Davis L Rose DP Hazard H Howard-McNatt M Adkins F Partin J Hobbs G Triple-negative breast cancer and obesity in a rural Appalachian population Cancer Epidemiol Biomarkers Prev 2008 17 3319 3324 10.1158/1055-9965.EPI-08-0544 19064545 PMC4665096

[B26] Solin LJ Hwang W Vapiwala N Outcome after breast conservation treatment with radiation for women with triple-negative early-stage invasive breast carcinoma Clin Breast Cancer 2009 9 96 100 10.3816/CBC.2009.n.018 19433390

[B27] NCCN Practice Guidelines in Oncology – V.2. – BINV 8 http://http//www.nccn. org/professionals/physician_gls/f_guidelines.asp

[B28] Hayes DF Thor AD Dressler LG Weaver D Edgerton S Cowan D Broadwater G Golstein LJ Martino S Ingle JN Henderson IC Norton L Winer EP Hudis CA Ellis MJ Berry DA HER2 and response to paclitaxel in node-positive breast cancer N Engl J Med 2007 357 1496 1506 10.1056/NEJMoa071167 17928597

[B29] Loesch D Greco F O'Shaughnessy J A randomized, multicenter, phase III trial comparing regimens of doxorubicin + cyclophosphamide (AC) followed by paclitaxel to doxorubicin + paclitaxel (AP) followed by weekly paclitaxel (wP) as adjuvant therapy for patients with high-risk, operable breast cancer J Clin Oncol 2007 25 Suppl 18 Abstract 517

[B30] Hudis C Citron M Berry D Five year follow-up of INT C9741: dose-dense (DD) chemotherapy (CRx) is safe and effective [abstract 41] Breast Cancer Res Treat 2005 94 Suppl 1 S20

[B31] Rouzier R Perou CM Symmans WF Ibrahim N Cristofanilli M Anderson K Hess KR Stec J Ayers M Wagner P Morandi P Fan C Rabiul I Ross JS Hortobagyi GN Pusztai L Breast cancer molecular subtypes respond differently to preoperative chemotherapy Clin Cancer Res 2005 11 5678 5685 10.1158/1078-0432.CCR-04-2421 16115903

[B32] Carey LA Dees EC Sawyer L Gatti L Moore DT Collichio F Ollila DW Sartor CI Graham ML Perou CM The triple negative paradox: primary tumor chemosensitivity of breast cancer subtypes Clin Cancer Res 2007 13 2329 2334 10.1158/1078-0432.CCR-06-1109 17438091

[B33] Baselga J Zambetti M Llombart-Cussac A Manikhas G Kubista E Steger GG Makhson A Tjulandin S Ludwig H Verill M Ciruelos E Egyhazi S Xu LA Zerba KE Lee H Clark E Galbraith S Phase II genomics study of ixabepilone as neoadjuvant treatment for breast cancer J Clin Oncol 2009 27 526 534 10.1200/JCO.2007.14.2646 19075286

[B34] Kassam F Enright K Dent R Dranitsaris G Myers J Flynn C Fralick M Kumar R Clemons M Survival outcomes for patients with metastatic triple-negative breast cancer: implications for clinical practice and trial design Clin Breast Cancer 2009 9 29 33 10.3816/CBC.2009.n.005 19299237

[B35] Thomas ES Gomez HL Li RK Chung HC Fein LE Chan VF Jassem J Pivot XB Klimovsky JV de Mendoza FH Xu B Campone M Lerzo GL Peck RA Mukhopadhyay P Vahdat LT Roché HH Ixabepilona plus capecitabine for metástasis breast cáncer progressing after antracyclines and taxane treatment J Clin Oncol 2007 25 5210 5217 10.1200/JCO.2007.12.6557 17968020

[B36] Roche H Li RK Ro J Ixabepilone plus capecitabine improves progression free survival in patients with metastatic breast cancer resistant to taxanes: a pooled analysis from two phase III trials Cancer Res 2009 69 Suppl 2 Abstract 2015

[B37] Farmer H McCabe N Lord CJ Tutt AN Jonson DA Richardson TB Santatosa M Dillon KJ Hickson I Knights C Martin NM Jackson SP Smtih GC Ashworth A Targeting the DNA repair defect in BRCA1 mutant cells as a therapeutic strategy Nature 2005 434 917 921 10.1038/nature03445 15829967

[B38] Bhattacharyya A Ear US Koller BH Weichselbaum RR Bishop DK The breast cancer susceptibility gene BRCA1 is required for subnuclear assembly of Rad5 and survival following treatment with the DNA cross-linking agent cisplatin J Biol Chem 2000 275 23899 23903 10.1074/jbc.C000276200 10843985

[B39] Turner N Tutt A Ashworth A Hallmarks of 'BRCAness' in sporadic cancers Nat Rev Cancer 2004 4 814 819 10.1038/nrc1457 15510162

[B40] Garber JF Richardson A Harris LN Neo-adjuvant cisplatin (CDDP) in 'triple-negative' breast cancer (BC) [abstract 308] Breast Cancer Res Treat 2006 100 Suppl 1 S32

[B41] Sirohi B Arnedos M Popat S Ashley S Nerurkar A Walsh G Johnston S Smith IE Platinum-based chemotherapy in triple negative breast cancer Annals Oncol 2008 19 1847 1852 10.1093/annonc/mdn395 18567607

[B42] Yi S Uhm J Cho E Clinical outcomes of metastatic breast cáncer patients with triple-negative phenotype who received platinum-containing chemotherapy [abstract 1086] J Clin Oncol 2008 26 Suppl 15 43s

[B43] Chia JW Ang P See H Triple-negative metastasic/recurrent breast cancer: treatment with paclitaxel/carboplatin combination chemotherapy [abstract 1086] J Clin Oncol 2007 25 Suppl 18 53s

[B44] Tan AR Swain SM Therapeutic strategies for triple-negative breast cancer Cancer J 2008 14 343 351 10.1097/PPO.0b013e31818d839b 19060597

[B45] Goffin JR Straume O Chappuis PO Brunet JS Bégin LR Hamel N Wong N Akslen LA Foulkes WD Glomeruloid microvascular proliferation is associated with p53 expression, germline BRACA1 mutations and an adverse outcome following breast cancer Br J Cancer 2003 89 1031 1034 12966421 10.1038/sj.bjc.6601195 PMC2376955

[B46] Miller K Wang M Gralow J Dickler M Cobleigh M Perez EA Shenkier T Cella D Davidson NE Paclitaxel plus bevacizumab versus paclitaxel alone for metastatic breast cancer N Engl J Med 2007 357 2666 2676 10.1056/NEJMoa072113 18160686

[B47] Miles D Chan A Romieu G Randomized, doubled-blind, placebo-controlled, phase III study of bevacizumab (BV) with docetaxel (D) or docetaxel with placebo (PL) as first-line therapy for patients with locally recurrent or metastatic breast cancer (mBC): AVADO [abstract LBA 1011] J Clin Oncol 2008 26 Suppl 15 1008s

[B48] Robert N Dieras V Glaspy J Ribbon-1: randomized, double-blind, placebo-controlled, phase III trial of chemotherapy with or without bevacizumab (B) for first-line treatment of HER2-negative locally recurrent or metastatic breast cancer (MBC) J Clin Oncol 2009 27 Suppl 15 Abstract 1005 10.1200/JCO.2010.28.0982 21383283

[B49] Mendel DB Laird AD Xin X Louie SG Christensen JG Li G Schreck RE Abrams TJ Ngai TJ Lee LB Murray LJ Carver J Chan E Moss KG Haznedar JO Sukbuntherng J Blake RA Sun L Tang C Miller T Shirazian S McMahon G Cherrington JM In vivo antitumor activity of SU11248, a novel tyrosine kinase inhibitor targeting vascular endothelial growth factor and plateletderived growth factor receptors: determination of a pharmacokinetic/pharmacodinamic relationship Clin Cancer Res 2003 9 327 337 12538485

[B50] Abrams TJ Lee LB Murray LJ Pryer NK Cherrington JM SU 11248 inhibits KIT and platelet-derived growth factor receptor beta in preclinical models of human small cell lung cancer Mol Cancer Ther 2003 2 757 766 12748309

[B51] Murray LJ Abrams TJ Long KR Ngai TJ Olson LM Hong W Keast PK Brassard JA O'Farrell AM Cherrington JM Pryer NK SU 11248 inhibits tumor growth and CSF-1R-dependent osteolysis in an experimental breast cancer bone metastasis model Clin Exp Metastasis 2003 20 757 766 10.1023/B:CLIN.0000006873.65590.68 14713109

[B52] Burstein HJ Elias AD Rugo HS Cobleigh MA Wolff AC Eisenberg PD Lehman M Adams BJ Bello CL DePrimo SE Baum CM Miller KD Phase II study of sunitinib malate, an oral multitargeted tyrosine kinase inhibitor, in patients with metastatic breast cancer previously treated with an anthracycline and a taxane J Clin Oncol 2008 26 1810 1816 10.1200/JCO.2007.14.5375 18347007

[B53] Barrios C Liu M Lee S A phase III randomized trial of sunitinib versus capecitabine in patients with previously treated HER 2 negative advanced breast disease (SUN 1107) [abstract 46] Proceedings of the San Antonio Breast Cancer Symposium 2009 32nd Annual San Antonio Breast Cancer Symposium http://www.sabcs.org

[B54] Baselga J Roche H Costa F SOLTI 0701: a multinacional double-blind, randomized phase 2b study evaluating the efficacy and safety of sorafenib compared to placebo when administered in combination with capecitabine in patients with locally advanced or metastatic breast cancer [abstract 45] Proceedings of the San Antonio Breast Cancer Symposium 2009 32nd Annual San Antonio Breast Cancer Symposium http://www.sabcs.org

[B55] Gradishar W Kaklamani V Prasad Sahoo T A double-blind, randomized, placebo-controlled, phase 2b study evaluating the efficacy and safety of sorafenib in combination with paclitaxel as a first line therapy in patients with locally recurrent or metastatic breast cancer [abstract 44] Proceedings of the San Antonio Breast Cancer Symposium 2009 32nd Annual San Antonio Breast Cancer Symposium http://www.sabcs.org

[B56] Fong PC Boss DS Yap TA Tutt A Wu P Mergui-Roelvink M Mortimer P Swasiland H Lau A O'Connor MJ Ashworth A Carmichael J Kaye SB Schellens JH de Bono JS Inhibition of poly(ADP-ribosa) polymerase in tumors from BRCA mutation carriers N Engl J Med 2009 361 123 134 10.1056/NEJMoa0900212 19553641

[B57] Bryant HE Schultz N Thomas HD Parker KM Flower D Lopez E Kyle S Meuth M Curtin NJ Helleday T Specific killing of BRCA2-deficient tumours with inhibitors of poly (ADP-ribose) polymerase Nature 2005 434 913 917 erratum Nature 2007, 447:346 10.1038/nature03443 15829966

[B58] Evers B Drost R Schut E de Bruin M van der Burg E Derksen PW Holstege H Liu X van Drunen E Beverloo HB Smith GC Martin NM Lau A O'Connor MJ Jonkers K Selective inhibition of BRCA-2 deficient mammary tumor cell growth by AZD2281 and cisplatin Clin Cancer Res 2008 14 3916 3925 10.1158/1078-0432.CCR-07-4953 18559613

[B59] Tutt A Robson M Garber JE Domchek S Audeh MW Weitzel JN Friedlander M Carmichael J Phase II trial of the oral PARP inhibitor olaparib-deficient advanced breast cancer J Clin Oncol 2009 27 Suppl 15 CRA501

[B60] O'Shaughnessy J Osborne C Pippen J Yoffe M Patt D Monaghan G Rocha C OssovsKaya V Sherman B Bradley C Efficacy of BSI-201, a poly (ADP-ribose) polymerase-1 (PARP1) inhibitor, in combination with gemcitabine/carboplatin (G/C) in patients with metastatic triple-negative breast cancer (TNBC): results of a randomized phase II trial J Clin Oncol 2009 27 Suppl 15 Abstract 3

[B61] Corkery B Crown J Clynes M O'Donavan N Epidermal growth factor receptor as a potential therapeutic target in triple negative breast cancer Ann Oncol 2009 20 862 867 10.1093/annonc/mdn710 19150933

[B62] Pal SK Mortimer J Triple negative breast cancer: novel therapies and new directions Maturitas 2009 63 269 274 10.1016/j.maturitas.2009.06.010 19632796

[B63] Gholam D Chebib A Hauteville D Bralet MP Jasmin C Combined paclitaxel and cetuximab archieved a major response on the skin metastases of a patient with epidermal growth factor receptor-positive, estrogen receptor-negative, progesterone receptor-negative and human epidermal growth factor receptor-2-negative (triple-negative) breast cancer Anticancer Drugs 2007 18 835 837 10.1097/CAD.0b013e3280adc8e0 17581308

[B64] Carey L Rugo H Marcom S TBCRC 001: EGFR inhibition with cetuximab added to carboplatinum in metastatic TNBC (basal like) J Clin Oncol 2008 26 suppl 15 Abstract 1009

[B65] O'Shaughnessy J Weskstein D Vukelja S Results of a randomized phase II study of weekly irinotecan/carboplatin with or without cetuximab in patients with metastatic breast cancer Breast Cancer Res Treat 2007 106 Suppl 1 S32

[B66] Clinical Trials http://www.ClinicalTrials.gov

[B67] Baselga J Albanell J Ruiz A Lluch A Gascón P Guillém V González S Sauldea S Marimón I Tabernero JM Koehler MT Rojo F Phase II and pharmacodynamic study of Gefitinib in patients with advanced breast cancer J Clin Oncol 2005 23 5323 5333 10.1200/JCO.2005.08.326 15939921

[B68] Nielsen TO Hsu FD Jensen K Cheang M Karaca G Hu Z Hermandez-Boussard T Livasy C Cowen D Dressler L Akslen LA Ragaz J Gown AM Gilks CB van de Rijn M Perou CM Immnunohistochemical and clinical characterization of the basal like subtype of invasive breast carcinoma Clin Cancer Res 2004 10 5367 5374 10.1158/1078-0432.CCR-04-0220 15328174

[B69] Conlin AK Seidman AD Beyond cytotoxic chemotherapy for the first line treatment of HER 2-negative, hormone insensitive metastatic breast cancer: current status and future opportunities Clin Breast Cancer 2008 8 215 223 10.3816/CBC.2008.n.024 18650151

[B70] Verbeek B Vroom T Adriansen-Slot S Ottenhoff-Kalff AE Geertzema JG Hennipman A Rijksen G c-Src protein expression is increased in human breast cancer. An immunohistochemical and biochemical analysis J Pathol 1996 180 383 388 10.1002/(SICI)1096-9896(199612)180:4<383::AID-PATH686>3.0.CO;2-N 9014858

[B71] Finn RS Dering J Ginther C Wilson CA Glaspy P Tchekmedyian N Slamon DJ Dasatinib, an orally ative small molecule inhibitor of both the src and abl kinases, selectively inhibits growth of basal-type triple negative breast cancer cell lines growing in vitro Breast Cancer Res Treat 2007 105 319 326 10.1007/s10549-006-9463-x 17268817

[B72] Finn R Bengala C Ibrahim N Phase II trial of dasatinib in triple-negative breast cancer: results of study CA Cancer Res 2009 69 Suppl 2 Abstract 3118

[B73] Saal LH Holm K Maurer M Merneo L Su T Wang X Yu JS Malmström PO Mansukhani M Enoksson J Hibshoosh H Borg A Parsons R PIK3CA mutations correlate with hormonoreceptors, node metastasis and ERBB2 and are mutually exclusive with PTEN loss in human breast carcinoma Cancer Res 2005 65 2554 2559 10.1158/0008-5472-CAN-04-3913 15805248

[B74] Ellard SL Clemons M Gelmon KA Norris B Kennecke H Chia S Pritchard K Eisen A Vandenberg T Taylor M Sauerbrei E Michaeli M Huntsman D Walsh W Olivo M McIntosh L Seymour L Randomized phase II study comparing two schedules of everolimus in patients with recurrent/metastatic breast cancer: NCIC clinical trials group IND J Clin Oncol 2009 27 4536 4541 10.1200/JCO.2008.21.3033 19687332

[B75] Whitesell L Mimmaugh E De Costa B Myers CE Neckers LM Inhibition of HSP90-pp60v-src heteroprotein complex formation by benzoquinone ansamycins: essential role for stress proteins in oncogenic trasnformation Proc Natl Acad Sci U S A 1994 91 8324 8328 8078881 10.1073/pnas.91.18.8324 PMC44598

[B76] Modi S Heat shock protein 90 inhibition: a novel strategy for the treatment of HER2-positive breast cancer Proceedings of the San Antonio Breast Cancer Symposium 2009 32nd Annual San Antonio Breast Cancer Symposium http://www.sabcs.org

[B77] Caldas-Lopez E Cerchietti L Ahn J Clement CC Robles AI Rodina A Moulick K Taldone T Gozman A Guo Y Wu N de Stanchine E White J Gross SS Ma Y Varticovski L Melnick A Chiosis G Hsp90 inhibitor PU-H71, a multimodal inhibitor of maliganancy induces complete responses in triple negative breast cancer models Proc Natl Acad Sci U S A 2009 106 8368 8373 19416831 10.1073/pnas.0903392106 PMC2688867

[B78] Giatromanolaki A Sivridis E Fiska A Koukourakis MI The CD44^+^/CD24^–^ phenotype relates to triple negative state and unfavorable prognosis in breast cancer patients Med Oncol Epub ahead of print 10.1007/s12032-010-9530-3 20405247

